# Stochastic Oscillations in Genetic Regulatory Networks: Application to Microarray Experiments

**DOI:** 10.1155/BSB/2006/59526

**Published:** 2006-10-03

**Authors:** Simon Rosenfeld

**Affiliations:** 1 Division of Cancer Prevention, Biometry Research Group, National Cancer Institute, Bethesda, MD 20892, USA

## Abstract

We analyze the stochastic dynamics of genetic regulatory networks using a system of nonlinear differential equations. The system of -functions is applied to capture the role of RNA polymerase in the transcription-translation mechanism. Using probabilistic properties of chemical rate equations, we derive a system of stochastic differential equations which are analytically tractable despite the high dimension of the regulatory network. Using stationary solutions of these equations, we explain the apparently paradoxical results of some recent time-course microarray experiments where mRNA transcription levels are found to only weakly correlate with the corresponding transcription rates. Combining analytical and simulation approaches, we determine the set of relationships between the size of the regulatory network, its structural complexity, chemical variability, and spectrum of oscillations. In particular, we show that temporal variability of chemical constituents may decrease while complexity of the network is increasing. This finding provides an insight into the nature of "functional determinism" of such an inherently stochastic system as genetic regulatory network.

## 

[1,2,3,4,5,6,7,8,9,10,11,12,13,14,15,16,17,18,19,20,21,22,23,24,25,26,27,28,29,30,31,32,33,34,35,36,37,38]
